# Ordinary people think merely of spending time, but schizotypy perceives time more accurately

**DOI:** 10.1177/17470218251349480

**Published:** 2025-06-04

**Authors:** Matthew Hopkins, Phil Reed, Irene Reppa, Paul Hitchcott

**Affiliations:** 1 Department of Psychology, University of Northampton, UK; 2 School of Psychology, Swansea University, UK

**Keywords:** Time Perception, scalar expectancy theory, psychophysics, schizophrenia, schizotypy

## Abstract

Duration judgement is a central component of cognitive functioning; however, a substantial body of evidence suggests that time perception is impaired in individuals with schizophrenia and schizotypy, respectively. Conclusions about the similar aetiology of both are constrained by empirical evidence with no evidence about the performance of schizotypy in the temporal estimation task. For the first time, a temporal estimation task examined the impact of schizotypy on both the retrospective and prospective paradigms for visual stimuli. The task involved subjects estimating one of three durations (15 s, 30 s, or 45 s) of a kitten video in either a retrospective or prospective paradigm in Experiment 1 and a video of the River Nene in Northampton, United Kingdom, in Experiment 2. Critical findings that emerged from this study are that high schizotypy subjects appear to have a greater degree of accuracy estimating durations, which is driven by the context of the stimulus. This finding implies that the pacemaker/accumulator component of scalar expectancy theory can be used to further explore timing deficits in schizophrenic subjects and might further imply that timing deficits in schizophrenia are driven by attentional deficits.

‘Your claim to superiority depends on the use you have made of your time and experience’ (Brontë, 1847). The muses of Charlotte Brontë in the classic *Jane Eyre* evoke the importance of the perception of time for the human experience (Matthews & Meck, 2014). Therefore, any deficits to the perception of time are likely to have an impact on everyday life, necessitating further research into the perception of time. Many psychopathologies are associated with deficits in time perception, including depression (Thönes & Oberfeld, 2015), anxiety (Bar-Haim et al., 2010) and Schizophrenia (Thoenes & Oberfeld, 2017). Of these, Schizophrenia is of most interest; however, to explore the perception of time in schizophrenia, a robust theoretical model of time perception is necessary.

Beginning with Treisman (1963), researchers have attempted to provide a theoretically robust cognitive model of the perception of time, motivated by the fact that unlike traditional perceptual senses, the perception of time does not have a dedicated biological organ (Grondin & Laflamme, 2015), though some authors contest that time perception is driven by so-called neutral oscillators (Treisman, 2013). One of the most popular and successful models of the perception of time is the scalar expectancy theory (SET) developed by Gibbon (1977). This model of the perception of time features several mechanistic components: the first is an attentionally driven pacemaker which emits Poisson-distributed pulses (Meck & Church, 1984); the pulses traverse towards an attentional ‘switch’. This switch, it is argued is driven by attentional resources (cf. Zakay & Block, 1996). Once the pulses have ‘passed’ the switch, the pulses accumulate and are stored at the accumulator though some pulses can be ‘lost’ if there is variability in the switch (Zakay & Block, 1996). From the accumulator, important pulses (i.e. such as subjects being trained on a length of a duration) are passed and subsequently stored in reference memory with subsequent pulses stored in working memory. The durations stored in both Reference and Short-Term memory are then compared, and a decision is made on whether the durations held in Working Memory matches durations that are held in Reference memory (Gibbon, 1977). SET has been used since the late 1970s to model time perception and has provided useful empirical evidence on how human and non-human subjects perceive time. SET has been used as a theoretical model for schizophrenia and schizotypy (i.e. Reed & Randell, 2014; Carroll et al., 2008), respectively. Despite the successes of SET, there are difficulties in applying SET to investigating the Perception of Time in Schizophrenia, which depend on many factors, including the length of durations investigated (i.e. so-called ‘critical timings’), as well as the paradigm use (i.e. prospective or retrospective). Also, the assumptions of SET have been subjected to rigorous debate, particularly around the mechanistic properties of the pacemaker–accumulator.

A critique of SET is that the theory appears to be unfalsifiable due to several assumptions (Buhusi & Oprisan, 2013). One of these key assumptions if that there is a linear relationship between time and the number of ticks emitted from the pacemaker meaning that any deviation of this relationship leads to an invariance in the perception of time (Gibbon, 1977). Therefore, several researchers have proposed additional mechanistic properties of the pacemaker–accumulator model, including the neural oscillation model of time perception (Treisman et al., 1994). For example, it has been tenuously argued by Treisman et al. (1994) that the perception of time is related to several temporal pacemakers, within the alpha band (i.e.8 to 13 Hz ); and that accurate representation of the perception of time is dependent upon these pacemakers having a sequence of parallel harmonically related frequencies (Treisman et al., 1994). Any deviancy within the frequencies of these pacemakers could lead to deficits in the perception of time, in which faster alpha rates could produce overestimation of durations; whilst slower alpha rates are linked to underestimation of durations, which is especially pertinent to schizophrenia, given that patients with schizophrenia show abnormalities in neural oscillations (Yeum & Kang, 2018). Despite these two mechanistic approaches to the pacemaker–accumulator model, there are several pertinent issues around critical time and paradigm which researchers must consider when investigating time perception.

Within the timing literature – and particularly with respect to research into Schizophrenia – subsecond durations (i.e. durations <1,000ms ) are often researched (Reed & Randell, 2014; Carroll et al., 2008). Subsecond durations are thought to be controlled by the basal ganglia and sensory responses (Rammsayer, 1993). Conversely, suprasecond durations (i.e. durations >1,000ms ) are thought to be controlled by higher cognitive functions, such as attention and memory (Grondin, 2014). These critical timing durations restrict researchers on what type of tasks can be administered (cf., Grondin, 2014); as well as paradigm can be investigated. A further issue with suprasecond durations is that subjects can employ chronometric counting strategies, which could lead to unaccounted for variance within data. To alleviate this, researchers often employ subsecond durations to limit chronometric counting (Riemer et al., 2022). The choice of critical timing value also affects which paradigm researchers can investigate. With respect to paradigm, there are two types within time perception research that researchers must consider, which are prospective and retrospective timing, respectively. In the prospective paradigm, the subject is aware that they are conducting a timing task and can draw on cognitive resources, such as attention and working memory to determine how long a duration lasted (Zakay & Block, 1996). Conversely, the retrospective paradigm is where subjects are not aware they are in a timing task and consequently, must draw on reference memory to determine how long a duration lasts (Klapproth, 2007; Zakay & Block, 1996).

In terms of investigating time perception in schizophrenia, few studies focus on suprasecond duration or the retrospective paradigm representing a methodological gap in the literature. Furthermore, since most researchers focus on the prospective paradigm, with subsecond durations, the underlying assumption is that any reported deficits in timing are due to the pacemaker/accumulator model and consequently, attention, which further illustrates a gap in the timing literature as most studies focus on attentional deficits in schizophrenia. However, there are theoretical and methodological challenges in using schizophrenic subjects, which have contributed to the somewhat contradictory findings in timing deficits found within this population.

Deficits in the perception of time are a prominent feature of schizophrenia (e.g. Carroll et al., 2008; Elvevåg et al., 2003; Roy et al., 2012; Snowden & Buhusi, 2019) and psychometrically defined schizotypy (Reed & Randell, 2014); however, the direction of these timing deficits is often contradictory (Carroll et al., 2009; Elvevåg et al., 2003; Ueda et al., 2018), with some studies reporting underestimation of durations, and others overestimation of durations in the subsecond domains with respect to schizophrenia. Several potential reasons underlying these differences in time perception have been posited, including working memory, reference memory and attentional mechanisms, respectively (Thoenes & Oberfeld, 2017), but the precise cognitive mechanisms that give rise to temporal dysfunction in schizophrenia remain unclear (Thoenes & Oberfeld, 2017). These cognitive mechanisms are further complicated by the fact that subsecond durations are likely to draw on automatic processes, whilst suprasecond durations are likely to draw on higher cognitive durations (Carroll et al., 2009; Reed & Randell, 2014). The issue is further compounded by the fact that time perception can be studied either prospectively or retrospectively, of which both paradigms draw on different cognitive mechanisms that are of relevancy to schizophrenia (Reed & Randell, 2014). These contradictory findings in schizophrenia could be explained by typical antipsychotics (e.g. Haloperidol, Thioridazine etc.), which are known to impair performance of the cognitive mechanisms that are thought to be responsible for time perception. For example, several studies (i.e. Rammsayer, 1989, 1993) have demonstrated that dopaminergic antagonists often lead to deficits in time perception (Goldstone et al., 1979). Therefore, it could be argued that the contradictory findings in the literature, with respect to schizophrenia, are the result of a lack of control of subjects’ use of medication (Reed & Randell, 2014); implying that schizotypy might be useful for investigating timing deficits in schizophrenia, given the converging evidence of a similar aetiology between schizophrenia and schizotypy, respectively.

Schizotypy has provided a useful model for schizophrenia (e.g. Fenner et al., 2020; Reed et al., 2008; Tsakanikos & Reed, 2005), given that it is contended that schizotypy is the expression of schizophrenia-like behavioural traits in the non-clinical population (Lenzenweger, 2006). This contention is given further impetus by the finding that schizophrenic patients and subjects with high schizotypy scores show similar performance across many cognitive tasks (Dagnall & Parker, 2009; Lee et al., 2006; Reed & Randell, 2014; Tsakanikos & Reed, 2005; see Siddi et al., 2017 for review). Furthermore, evidence suggests that memory deficits in schizotypy are similar to those of schizophrenia (Vollema & Postma, 2002), which is relevant to time perception, particularly, retrospective time perception demonstrating that schizotypy might present opportunities in studying the aetiology of time perception in schizophrenia.

Despite the opportunities that schizotypy may present for furthering a theoretical understanding of timing processes in schizophrenia, there are many empirical gaps in the knowledge base. Importantly, when tested in timing tasks, those with high schizotypy scores sometimes have more accurate temporal judgement, tending to show less underestimation of time, at least at subsecond durations (Reed & Randell, 2015), which contrasts with studies of medicated schizophrenic subjects (e.g. Carroll et al., 2009). An explanation for these contradictory findings could be that subjects with schizophrenia (and the schizophrenia spectrum-like personality characteristics, such as schizotypy) that take part in tasks where attentional load is low (i.e. such as a temporal bisection task) show greater impairment than controls. Conversely, when attentional demands are high (i.e. a context-rich stimulus), schizophrenic subjects are more likely to pay more attention (Ducato et al., 2008) to the stimulus. In the context of SET, this finding could be explained by the contextual nature of a stimulus determining whether subjects will perceive a duration accurately or not. However, most studies investigating time perception in both schizophrenia and schizotypy are focused on subsecond durations, and within the prospective paradigm, meaning there are important theoretical considerations in terms of experimental design, given that subsecond durations are largely thought to be controlled by automatic cognitive processes (Grondin, 2010).

As discussed, subsecond durations are thought to be controlled by automatic and sensory processes (Thibault et al., 2013); while suprasecond durations are controlled by higher cognitive mechanisms (Grondin, 2014). Furthermore, prospective timing is based on attention and working memory, while retrospective timing is presumably based on reference memory (Klapproth, 2007; Zakay & Block, 1996), which demonstrates there are distinct cognitive mechanisms that drive time perception based on both task and duration, respectively. The majority of studies that have investigated time perception in schizophrenia and schizotypy are typically prospective and subsecond in nature (i.e. Temporal Bisection or Generalisation). A difficulty is that these tasks often give contradictory results from one another (cf. Carroll et al., 2008; Reed & Randell, 2014), making it difficult to delineate the nature and/or source of timing deficits for schizophrenia therefore, the Temporal Estimation tasks presents a unique task, for these populations, to examine suprasecond and retrospective time perception (Klapproth, 2007) thereby focusing on several elements of time perception and different cognitive mechanisms.

Few studies have used the temporal estimation task for schizophrenia (Ueda et al., 2022), and none at the time of writing has explored retrospective timing in schizophrenia or schizotypy, respectively. An advantage of the temporal estimation task is that it can be used in both a retrospective and prospective manner, thereby allowing researchers to investigate both attentional and memory aspects of time perception, as well as higher cognitive mechanisms that might be driving time perception (Klapproth, 2007). Under the prospective paradigm, the subject makes a duration estimation under the premise that timing is relevant (Klapproth, 2007; Zakay & Block, 2004); therefore, they are paying attention to the duration. Conversely, in the retrospective paradigm, subjects are not aware that duration is relevant until after the task (Klapproth, 2007), meaning they must draw on previously encoded information. Retrospective timing depends on remembered durations (Zakay & Block, 2004), thereby mapping onto memory (Klapproth, 2007), which may be subjected to deficits for those with schizophrenia (Rouy et al., 2021) and schizotypy (Sahakyan & Kwapil, 2016). Alternatively, Pouthas and Perbal (2004) suggest that prospective timing depends on attentional processes (Zakay & Block, 2004) and processing difficulties (Zakay & Block, 2004). Again, these map onto higher cognitive mechanisms, such as working memory and attention, both of which are suggested to be impaired in schizophrenia (i.e. McCutcheon et al., 2023; Seabury & Cannon, 2020) and schizotypy (Haigh et al., 2023; Huang et al., 2021); all of which are difficult to investigate with the subsecond time perception tasks, such as bisection or generalization.

Given these considerations, the current study used the temporal estimation procedure from Klapproth (2007) to assess retrospective and prospective estimation for subjects with low and high levels of schizotypy to investigate whether the attentionally driven pacemaker/accumulator mechanisms of SET drive deficits in timing in schizophrenia by utilizing schizotypy. A further aim was to further document the similarities between schizotypy and schizophrenia by extension to this novel task. This is important as Reed and Randell (2014), using a temporal bisection task, found greater accuracy in high schizotypy whereas Carroll et al. (2009) found the opposite with schizophrenic subjects; however, both of these studies used (a) prospective timing tasks and (b) subsecond durations. Based on previous findings, and the argument that schizophrenics might pay more attention to concrete stimuli, this study will test the assumption that timing deficits are based on attentional deficits, caused by an errant pacemaker/accumulator, by conducting two investigations. The first will employ a context-heavy stimulus (i.e. a kitten video), the second will employ a context-neutral stimulus (i.e. the River Nene, at Northampton).

## Experiment 1

Experiment 1 will test the following hypotheses: $H_{1}$ : that high schizotypy subjects will underestimate durations, relative to low schizotypy, in all conditions (given the finding by Reed & Randell, 2014), $H_{2}$ : that there will be a difference in accuracy between high schizotypy and low schizotypy in that high schizotypy will be more accurate in identifying durations in both the retrospective and prospective paradigm, given the contextual richness of the stimuli (given the finding by Ducato et al., 2008). Finally, $H_{3}$ states that intersubject variability should be higher in high schizotypy, if timing deficits are the result of attentional deficits, as posited by the pacemaker/accumulator model (Gibbon, 1977).

### Method

#### Subjects

A total of 325 subjects (180 females; 145 males) were recruited via The School of Psychology’s subject pool (Swansea University) and the Prolific platform. The mean age of subjects was 34.74 (*SD* = 15.63; range 18–79) years. In terms of outliers, subjects whose reproductions <3s were excluded from the data set, given that this is likely that they were not paying attention to the study and one subject, who responded 240 s to the 30 s duration was also excluded. In total, 4.62% of the sample population were removed (or 15 subjects) leaving 310 subjects for the analysis stage. After the data were cleaned for outliers, the positive schizotypy group was split at the mean of 13.23(SD=7.12) There were a total of 150 subjects in the Low Schizotypy group ( M=6.87±3.25 ) and 160 subjects in the High Schizotypy group ( M=19.20±3.81 ). Subjects in the retrospective paradigm were not told that timing would be involved in the study; whilst those in the prospective paradigm were informed that they were partaking in a timing task. Subjects were paid a total of £1.10 for their participation.

#### Power

Due to a computational error, an incorrect power analysis was initially made; however, given the sample size was already known, a G*Power sensitivity analysis was conducted to determine the Smallest Effect Size of Interest (SMSI) with the available subject size of 325subjects, desired power (i.e. 80%) and significance level (α=.05) . For a Between-Subjects ANOVA, the analysis gave a Cohen’s f of f=0.17 which when converted into $\eta^{2}$ gave $\eta^{2}\;=\;.029$ , which implies that the study is sufficiently powered to detect a small effect size.

### Stimuli and measures

The experiment was designed in the Gorilla.sc programme [Gorilla Experiment Builder]. Subjects completed the experiment on their own personal computers, which controlled all experimental events, and recorded their data. Responses were made on the subject’s own computer keyboard.

#### Stimulus

In terms of the target stimuli, a video depicting a kitten (*Felis* catus) was used to employ a potentially positively valanced cue, given how important attention is throughout the task (Lane et al., 1999), a context-rich video was required. The same video was used, and truncated, to create the 15 s, 30 s and 45 s durations for the randomized experimental conditions. The video presentation was preceded by a black cross presented on a white screen, which was displayed for 500 ms. The interval between the cross disappearing and the video being played was 1,500 ms.

#### Oxford Liverpool Inventory of Feelings and Experiences – Brief (OLIFE(B))

The O-LIFE(B) scale is a 43-item self-report scale for measuring schizotypy traits in the general population (Mason et al., 2005). The scale comprises four distinct subscales, each of which maps onto specific elements of Schizophrenia. Unusual Experiences (UE) maps onto the positive symptomology (i.e. perceptual aberrations), Cognitive Disorganization (CD) maps onto the cognitive symptoms, (i.e. lack of concentration) Introvertive Anhedonia (Impulsive Nonconformity [IN] maps onto the negative symptomology (i.e. loss of pleasure) and IN maps onto a lack of self-control (Premkumar et al., 2020). The scale is based upon empirically observed structures of schizotypal traits and has good validity in the general population (Green et al., 2008; Mason et al., 2005). O-LIFE(B) has been used extensively in examining the effect of schizotypy on behaviours and cognitions, including (though less so) time perception (Reed & Randell, 2014; Tsakanikos & Reed, 2005).

Given that UE, CD and IN map onto several elements of Time Perception (i.e. perceptions and cognitions) it can be difficult to analyse each of the subscales individually and mapping these onto SET, which itself has several components that map onto each of these components elements from O-LIFE(B) (Reed & Randell, 2014), which could lead to contradictory findings (i.e. as in Reed & Randell, 2014). Whilst some authors suggest against using the sum of the Schizotypy O-LIFE scale (Mason et al., 2005); others have taken this approach (Reed, 2023), in which the summing of UN, CD and IN are thought to give a measure for positive schizotypy (Reed, 2023). Therefore, we summed UE, CD and IN, given that each of the constituency components of these subscales combine to influence time perception more globally as opposed to locally, this summed variable was termed time-dependent – as opposed to positive – schizotypy. The decision was also taken to remove the IA subscale from the sum. The rationale for excluding IA was that depression has an effect on the subjective flow of time (Thönes & Oberfeld, 2015), as well as the link between IA and depression (Premkumar et al., 2020), which could further complicate any potential findings. The internal reliability (Cronbach α ) for the current sample was α=.66 ., which is considered satisfactory (Taber, 2018). The mean value of the time-dependent schizotypy scales was 13.23±7.12 , with a range of 0to30 . Subsequently, those with a score <13 were classified as having a lower manifestation of schizotypy and those who had a score >13 were classified as having a higher manifestation of schizotypy. A mean split was used, given the converging evidence that a median split increases the risk of a Type II error (McClelland et al., 2015). By taking this approach, we could conduct a between-subjects ANOVA.

The alternative analysis would have been a Multivariate Multiple Regression, in which the 4 schizotypy subscales would have been used as predictor variables, and each of the 6 (i.e. prospective and retrospective 15 s, 30 s and 45 s, respectively) dependent variables (DVs) would have been outcome variables. Such an analysis would have been difficult to map onto SET. Furthermore, similar studies using this paradigm (i.e. Klapproth, 2007) typically use an ANOVA design, which is what the authors decided upon. Finally, given that Reed and Randell (2014) categorize the schizotypy groups into ‘Low and High’, we decided to use this approach in this study. Therefore, it was decided to sum the subscales and test the differences (i.e. ANOVA) between low and high schizotypy subjects in accordance with Reed and Randell (2014) and Klapproth (2007).

### Design

The experiment was a 2 × 2 × 3 between-subjects design with paradigm (prospective and retrospective), and schizotypy level (lower versus higher) and duration of the kitten video (15 s, 30 s and 45 s) as between-subject factors. The DV was the estimate that subjects made to one of the three presented durations. For intersubject variability, the mean value was the DV, and the ratio value between actual and estimated durations was the DV. Both the intersubject variables and the ratio values were calculated in accordance with Klapproth (2007).

### Procedure

**Figure 1.**
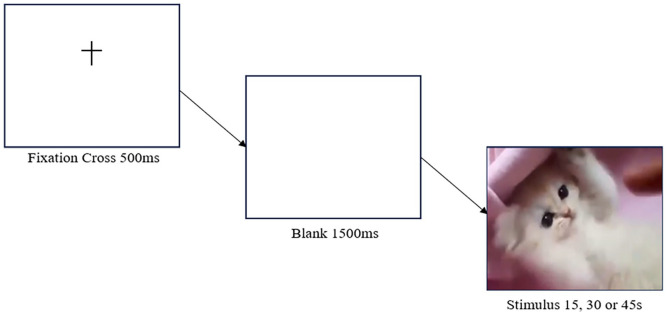
Schematic diagram of Experiment 1. *Note.* After the Subject Information Sheet and Consent, subjects were presented with a set of instructions. They then saw a fixation cross for 500ms , followed by a blank screen for 1,500 ms . They were then randomly presented with a single stimulus of a kitten video lasting for either 15s , 30s or 45s , respectively.

Subjects first read the Subject Information Sheet and were then asked to consent to the study. Once subjects had agreed, they were then randomized into either the retrospective condition (in which they were told that they were partaking in a visual perception task) or the prospective conditions (in which they were told that they were partaking in a time perception task). Once subjects were assigned to either the retrospective or prospective condition, subjects were further randomized into one of the three durations (i.e. 15 s, 30 s and 45 s). Subjects then read the task instructions. Once they had read the instructions, they were instructed to press the spacebar to continue, which started the experiment. A black cross on a white screen was then shown for 500ms . After the cross, a white screen was displayed for 1,500 ms . After the white screen, a temporal estimation task followed where subjects were asked to estimate, in seconds, how long the video lasted. Once they had entered a value via their keyboard (in seconds), they were instructed to press the spacebar and told that they have completed the experimental element of the experiment. Subjects then completed a basic demographics questionnaire and the O-LIFE Short questionnaire. A graphical representation of the experiment is shown in Figure 1.

### Data analysis

Despite the skew suggested by the violin plots, given that the sample for Experiment 1 is >200, the deviation of skew from normality will not make a substantive difference to the results (Tabachink & Fiddell, 2013). To further reinforce this, Kline (2011) argued that only skew values >3 could cause problems with data and given that all variables within Experimental 1 have skew values <3, the data are within acceptable parameters of skew (Kline, 2011). The data will be analysed with three Between-Subject Analysis of Variance (ANOVA) tests. The first ANOVA will have subject estimations as its DV, with Paradigm (prospective and retrospective), Duration (15s, 30s and 45s) and schizotypy (high and low) treated as Independent Variables (IV). The IVs in this study are Between-Subject factors; whilst the DV is the Within-Subject factor. The second ANOVA will use ratio as its DV, whilst the final ANOVA will utilize Intersubject Variability (InVa) as its DV. All significant Main Effects and Interactions will be further analysed using post hoc *t*-tests, using the Bonferroni-Corrected criteria.

## Results and discussion

**Figure 2.**
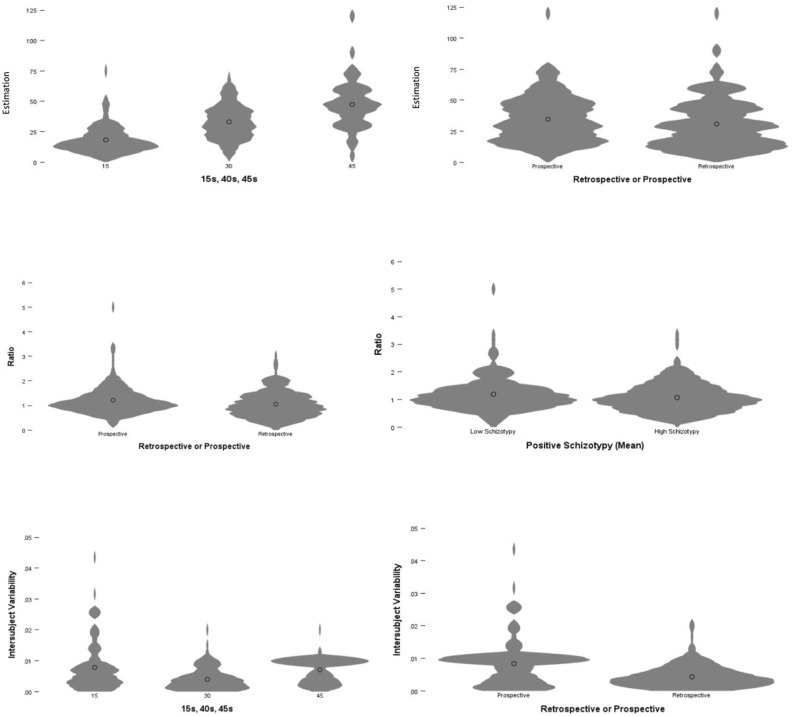
Results from Experiment 1. *Note.* On the top row, the violin plots show the main effect of duration (Panel 1); whilst in Panel 2, subjects are shown to generally underestimate durations in the retrospective paradigm, as opposed to the prospective paradigm. In row 2, it is shown that subjects (Panel 3) are more accurate in the retrospective paradigm, as opposed to the prospective paradigm. Interestingly, in Panel 4, High Schizotypy subjects were more accurate, irrespective of paradigm or duration, as indicated by a greater distribution of points around 1.00 . Finally, on the bottom row, it can be seen in Panel 5 that subjects’ intersubject variability significant for the 30s duration. Similarly, subjects had less intersubject variability in the retrospective paradigm, indicating they paid more attention to it.

To test $H_{1}$ , the estimation of subjects’ responses was tested. A three-factor between-subjects ANOVA with paradigm (Retrospective/Prospective) and schizotypy (low/high) and durations (15 s, 30 s and 45 s) as between-subject factors was conducted. As indicated in Figure 2 (Panel 1), there was a significant main effect of duration, F(2,298)= $90.819,p<.001,\;\;\;\eta^{2}=.379,\;95\%\;\mathrm{CI}$ [0.0000, 0.9284] and paradigm, $F\left(1,\;\;298\right)=5.698,\;\;p=.018,\;\eta^{2}=.019,$ [0.0004, 0.0594] however, there was no significant main effect of schizotypy, $F\left(1,\;\;298\right)=2.335,\;\;p=\;\;.128,\;\eta^{2}=.008,$ [0.0000,0.0390] . There were no significant interactions, (i.e. all $p_{s}>.1$ ). Given that the sensitivity analysis revealed that the SMSI was $\eta^{2}=.029$ , the main effect of duration $\left(\eta^{2}=.379\right)$ was above the threshold, implying the effect was detected robustly, despite the sample size. However, the main effect of paradigm ( $\eta^{2}=.019$ ) was below the threshold, implying the result should be interpreted with caution.

To further analyse the main effect of condition, Bonferroni-Corrected Post Hoc *t*-tests were conducted, in which the significance level after the correction was $\alpha =\frac{.05}{3}=.02$ . The mean difference (M=−39.06) between 15s(M=18.31;SD=11.28) and 30s (*M* = 47.37; SD=19.83) estimations were significant, t(204)= −13.020,p<.001,d=−1.815.95%CI[−2.14,1.49] , as was the mean difference (M=−14.81) between 15s(M=18.31;SD=11.28) and 30s (*M* = 33.12; *SD* =14.06) estimations, t(208)=−8.429,p<.001,d= −1.163,[−1.46,−0.87] , as was the mean difference (M=−14.25) between 30s(M=33.12;SD=14.06) and 45s(M=47.37;SD=19.83) was also significant t(202)=−5.940,p<.001,p=−.832,[−1.12,−0.55] , demonstrating that each of the durations were significantly different to one another, in accordance with Klapproth (2007).

To unpack the main effect of paradigm, Bonferroni-Corrected Post Hoc *t*-test tests were also conducted which showed that the mean difference (M=3.83) between retrospective (M=30.80;SD=19.95) and prospective (M=34.63;SD=18.64)) were significant, t(308)=1.744, p=.041,d=.20,95%CI[−0.025,0.421] . Consequently, the null hypothesis of $H_{1}$ thus cannot be rejected. Though, we note this main effect of paradigm suggesting that subjects were more likely, overall, to underestimate Retrospective Durations, as opposed to prospective Durations, as shown in Figure 2 (Panel 2).

To test $H_{2}$ , the ratio of estimations, relative to actual durations, was taken in which answers closer to 1 indicate greater accuracy in estimating the duration. There were significant main effects of paradigm, F(1,298)= $5.617,\;\;p=.018,\;\;\eta^{2}=.019,\;\;\;95\%\;\mathrm{CI}$ [0.0149, 0.1109] and schizotypy, $F\left(1,\;\;298\right)=3.943,\;p=.048.,\;\;\eta^{2}=\;\;.013,\;\;[0.0000,$ 0.0494] but not of durations, F(2,298)=2.804,p= $\;.062,\;\;\eta^{2}=.018,\;\;[0.0004,\;0.0548]$ . There were no significant interactions, (i.e. all $p_{s}>.1$ ). Similar to $H_{1}$ the significant main effect of paradigm $\left(\eta^{2}=\;.019\right)$ and the Schizotypy $\left(\eta^{2}=.013\right)$ should interpreted with caution, given the sensitivity analysis.

To further unpack the main-effects of paradigm, Bonferroni-Corrected post-hoc t tests were conducted, in which the mean difference (M=0.16) between the prospective M=1.21;SD=0.60) and retrospective (*M* = 1.04; SD=0.54) was significant t(308)=2.513,p= .006,d=0.29,95%CI[0.06,0.51] , implying that subjects were more accurate in the retrospective paradigm, than the prospective paradigm, as illustrated in Figure 2 (Panel 3). We used similar Bonferroni-Corrected *t*-test to unpack the main effect of schizotypy, in which the mean difference (M=0.12) between high schizotypy (M=1.07;SD=0.53) and low Sshizotypy (*M* = 1.2; SD=0.62) was significant, t(308)=1.904,p=.029., d=0.22,[−0.01,.0.44] , implying that high schizotypy was more accurate than low schizotypy, irrespective of durations and paradigms, respectively as illustrated in Figure 2 (Panel 4). Therefore $H_{2}$ can be accepted.

To test the final hypothesis $\left(H_{3}\right)$ , the intersubject variability of subjects’ responses was tested. A three-factor between-subjects ANOVA with paradigm (retrospective/prospective) and schizotypy (low/high) as between-subject factors, and duration (15 s, 30 s and 45 s) as a within-subject factor, was conducted on Intersubject Variability. There was a main effect of duration, $F\left(2,298\right)=18.324,\;\;p<.001,\;\eta^{2}=.110,\;95\%\;\mathrm{CI}\;$ [0.1095,0.1752], as shown in Figure 2 (Panel 5) and paradigm, $F\left(1,298\right)=50.784,\;p<.001,\;\eta^{2}=.146,\;[0.0792,\;$ 0.2186] , as show I Figure 2 (Panel 6) though, not of schizotypy, $F\left(1,298\right)\;=0.045,p=.831,\eta^{2}=.000,[0.0000,\;0.0064]$ . There was a significant interaction between condition and paradigm, $F\left(2,298\right)=13.904,p<.001,\;\eta^{2}=.085,\;[0.0317,$ 0.1467] , but no other interactions (all $p_{s}>.5$ ). The main effect of duration $\left(\eta^{2}=\;.110\right)$ and paradigm $\left(\eta^{2}=.146\right)$ should be interpreted with caution, in accordance with the sensitivity analysis.

A Bonferroni-Corrected *t*-test was conducted to unpack the main effect of paradigm, in which the mean difference (M=0.004) between retrospective (M=0.004; *SD* = 0.004) and prospective (M=0.008;SD=0.007) was significant, t(308)=6.534,p=.001,.0054,95%CI [0.51,0.92] as shown in Figure 2 (Panel 6) indicating that subjects demonstrated less variability in the retrospective paradigm than the prospective paradigm, implying that subjects were paying greater attention in the retrospective paradigm.

The main effect of condition was also unpacked by using a Bonferroni-Corrected t-test, in which the adjusted p -value was $p=\frac{.05}{3}=.02$ . We first compared the 15 s and 45 s durations of which the mean difference (M=0.00079 ) between 15 s (M=0.008;SD=0.008) and 45 s (M=0.007;SD=0.004) was not significant, t(204)=0.904,p=.183,d=0.13,95%CI[−0.15,0.40] . Secondly, the mean difference (M=0.004) between 15 s (M=0.008;SD=0.008) and 30 s (M=0.004;SD=0.004) was significant t(208)=p<.001,d=0.63, [0.35,0.90] . Finally, the mean difference (M=0.003 ) between 30 s (M=0.004;SD=0.004) and 45 s (0.007;SD=0.004) was significant, t(202)=−5.842,p<.001,d=−0.81, [−1.1,−0.53] implying that there was less subject variability in the 30 s condition than either 15 s or 45 s, respectively, meaning that overall, subjects were paying greater attention to the 30s duration, as demonstrated in Figure 2 (Panel 6).

Finally, to unpack the interaction between Paradigm and Duration, six Bonferroni-Corrected t-tests were first conducted to determine duration intersubject variability within paradigm, in which the adjusted significance level was $\alpha =\frac{.05}{6}=\;.008$ and these are reported in Table 1. For the Between-Paradigm comparison, three Bonferroni-Corrected *t*-tests were conducted, in which the adjusted significance level is given by $\alpha =\frac{.05}{3}=\;.012$ and is reported in Table 2 The post hoc comparisons revealed that within-paradigm, the 30 s intersubject variability was significantly lower compared to 15s and 45s durations, respectively, implying subjects were paying more attention to the 30s duration (in accordance to the main effect of condition). For Between-Paradigm comparisons, subjects’ intersubject variability was lower in the retrospective paradigm for both the 15s and 45s . Overall, subjects paid more attention to the 30s duration, but were paying greater attention in the retrospective paradigm meaning that we can retain the null hypothesis for $H_{3}$ , given the lack of interaction or main effect for schizotypy.

**Table 1.** Bonferroni-Corrected *t*-tests for Within-Paradigm in Experiment 1, in which the dependent variable is intersubject variability per duration.

Paradigm	Duration	Mean (*SD*)	Comparison	Difference	t(df)	p	d(95%CI)
Prospective	15 s	0.011,(0.009)	15sand45s	0.001	0.959(97)	.34	0.19,[−0.20,0.59]
	30 s	0.004,(0.005)	15sand30s	0.007	5.068,(101)	$.001^{*}$	0.99,[0.59,1.41]
	45 s	0.010,(0.000)	30sand45s	−0.006	−9.063,(96)	$.001^{*}$	−1.82,[−2.3,−1.35]
Retrospective	15 s	0.005,(0.004)	15sand45s	0.00008	0.100,(105)	.460	0.019,[−0.36,0.40]
	30 s	0.004,(0.003)	15sand30s	0.0005	0.767,(105)	.222	0.15,[−0.23,0.53]
	45 s	0.004,(0.004)	15sand30s	−0.0004	−0.716,(104)	.238	0.003,[−0.52,0.24]

*Note.* The asterisk (*) denotes a significant value. Furthermore, three Bonferroni-Corrected post hoc *t*-tests were first conducted for the prospective paradigm, followed by retrospective. After the Bonferroni-Correction was applied, the significance level was α=.00833 .

**Table 2.** Bonferroni-Corrected *t*-tests for Between-Paradigm in Experiment 1, in which the dependent variable is intersubject variability per duration.

Condition	Mean (*SD*)		Difference	t(df)	p	d(95%CI)
	Prospective	Retrospective				
15s	0.011,(0.009)	0.005,(0.004)	0.007	4.819(104)	$<.001^{*}$	0.93,[0.53,1.33]
30s	0.004,(0.005)	0.004,(0.003)	−0.00009	−0.126,(102)	.450	0.003,[−0.401,0.360] 0.003,[−0.401,0.360]
45s	0.010,(00001)	0.004,(0.004)	0.006	10.144,(98)	$<.001^{*}$	2.032[1.54,2.51]

*Note.* The asterisk (*) denotes a significant value. Furthermore, three Bonferroni-Corrected post hoc *t*-tests conducted for each condition comparing paradigm. After the Bonferroni-Correction was applied, the significance level was α=.01667 .

Therefore, for Experiment 1, we have confirmed the main hypotheses ( $H_{2}$ ) that high schizotypy subjects would be more accurate in identifying durations (i.e. as indexed by a ratio value of closer to 1) when the stimulus was contextually rich (in accordance with Ducato et al., 2008). In the context of SET by Gibbon (1977), this would imply that the schizotypy pacemaker/accumulator is more aroused by the concrete stimulus leading to a more accurate recall of durations (Treisman, 1963); whilst in the context of the neural oscillator, it could be argued that high schizotypy subjects have a disturbance in neutral oscillations, giving rise to more pulses accumulating at the accumulator. Such oscillation aberrations are observed in schizophrenic and schizotypal subjects (Zhou et al., 2021) though, contrary to opinion, this aberration does not lead to a deficit, but an advantage. To confirm the results of Experiment 1, a neutral stimulus needs to be used to determine whether this result can be explained by the contextually rich stimulus, which is the basis of Experiment 2.

## Experiment 2

Experiment 2 examined temporal estimations for low and high schizotypy similar to Experiment 1 however, this time, we used a contextually neutral stimulus in order to determine whether the finding in Experiment 1 that high schizotypy is more accurate in identifying durations, is the result of highly contextualized stimuli (Ducato et al., 2008). Given that it was argued in Experiment 1 that the lack of a pacemaker–accumulator deficit in high schizotypy was the result of them paying more attention to concrete stimuli (Ducato et al., 2008) than controls, it could be argued that the nature of the stimulus in Experiment 1 (i.e. a kitten video) led to high schizotypy achieving a higher degree of accuracy in identifying durations (Lenzenweger, 2006) given that the high schizotypy pacemaker–accumulator was more highly aroused (Treisman, 1963). Therefore, we aim to test several hypotheses that have been formulated based on the findings of Experiment 1. $H_{1}$ is that high schizotypy and low schizotypy will have no differences in duration estimations (i.e. they will neither underestimate or overestimate durations, relative to low schizotypy); $H_{2}$ is that high schizotypy will not show any deviation in accuracy compared to low schizotypy, given that we have used a contextually neutral stimulus; and finally $H_{3}$ states high schizotypy will not show any deviation in intersubject variability, given that the pacemaker–accumulator model should not be affected by a highly contextualized stimuli.

## Method

### Subjects

A new sample of 213 subjects (185 Female; 28 Male) were recruited at the University of Northampton as described in Experiment 1, but this time, they were awarded 4 credit points, as opposed to a monetary reward. Subjects were between 18 and 54 ( M=21.32±6.0) .

### Power

The sensitivity power analysis, discussed in Experiment 1, was conducted for Experiment 2, for 213 subjects. This yielded a Cohen’s f of f=0.21 which when converted to $\eta^{2}$ yielded a value of $\eta^{2}=.044$ ; implying that the study is sufficiently powered to detect a small effect size. Though, it is noted that Experiment 2 has less power to detect the effect than Experiment 1.

#### Stimuli and materials

The stimulus used in this study was a contextually neutral stimuli of the River Nene here at the University of Northampton, in the United Kingdom. A 60 s video was filmed by the principal researcher in 4K and then truncated to match the durations used in the study (i.e. 45 s, 30 s and 15 s, respectively).

Based on O-LIFE scores, we used the same strategy as Experiment 1 (i.e. sum the time-dependent subscales) and then conducted a mean split of the time-dependent schizotypy data after cleaning; of which for this sample M=18.32;SD=6.30 , in which the range was 2−31 ). There were 90 subjects in the Low Schizotypy group (M=12.72;SD=3.72) and 105 subjects in the High Schizotypy group (M=23.11;SD=3.45) .

### Design

Similar to Experiment 1, we used a between-subjects design, in which we had 2 schizotypy groups (low and high), 2 paradigms (prospective and retrospective) and 3 durations (15 s, 30 s and 45 s). Furthermore, subjects whose estimations were <3s and >240s were removed from the dataset (continuous with Experiment 1), meaning that 8.45% of the population sample was removed (or 18 subjects).

### Procedure

**Figure 3.**
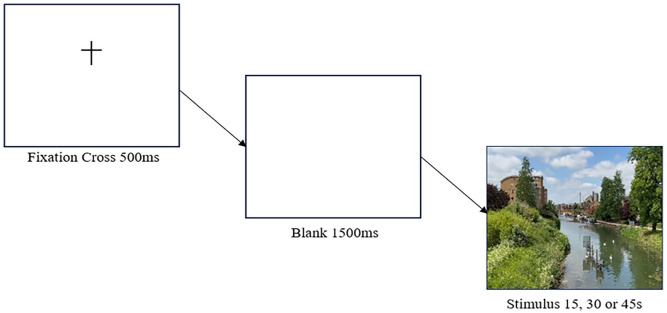
Schematic diagram of Experiment 2. *Note.* After the Subject Information Sheet and Consent, subjects were presented with a set of instructions. They then saw a fixation cross for 500 ms, followed by a blank screen for 1,500 ms. They were then randomly presented with a single stimulus of the River Nene in Northampton, United Kingdom, lasting for either 15s , 30s or 45s , respectively.

Similar to Experiment 1, subjects in the retrospective conditions were told that they were partaking in a visual perception task; whilst those in the prospective condition were told that they were partaking in a time perception task. In all cases, subjects were asked to pay close attention to the video, and all were presented with a set of instructions. Once they had read the instructions, they were instructed to press the spacebar to continue, which started the experiment, in which subjects received one of the three durations. A black cross on a white screen was shown for 500 ms. After the cross, a white screen was displayed for 1,500 ms. After the white screen, a temporal estimation task followed where subjects were asked to estimate, in seconds, how long the video lasted. Once they had entered a value via their keyboard, they were instructed to press the spacebar. Then completed a basic demographics questionnaire, and the O-LIFE questionnaire. A graphical representation of this design is shown in Figure 3.

### Data analysis

Similar to Experiment 1, the data will be analysed with three Between-Subject ANOVA tests. The first ANOVA will have estimations as its DV, with Paradigm (prospective and retrospective), duration (15 s, 30 s and 45 s) and schizotypy (high and low) treated as Independent Variables (IV). The IVs in this study are Between-Subject factors; whilst the DV is the Within-Subject factor. The second ANOVA will use ratio as its DV, whilst the final ANOVA will utilize InVa as its DV. All significant Main Effects and Interactions will be further analysed using post hoc *t*-tests, using a Bonferroni correction.

## Results and discussion

**Figure 4.**
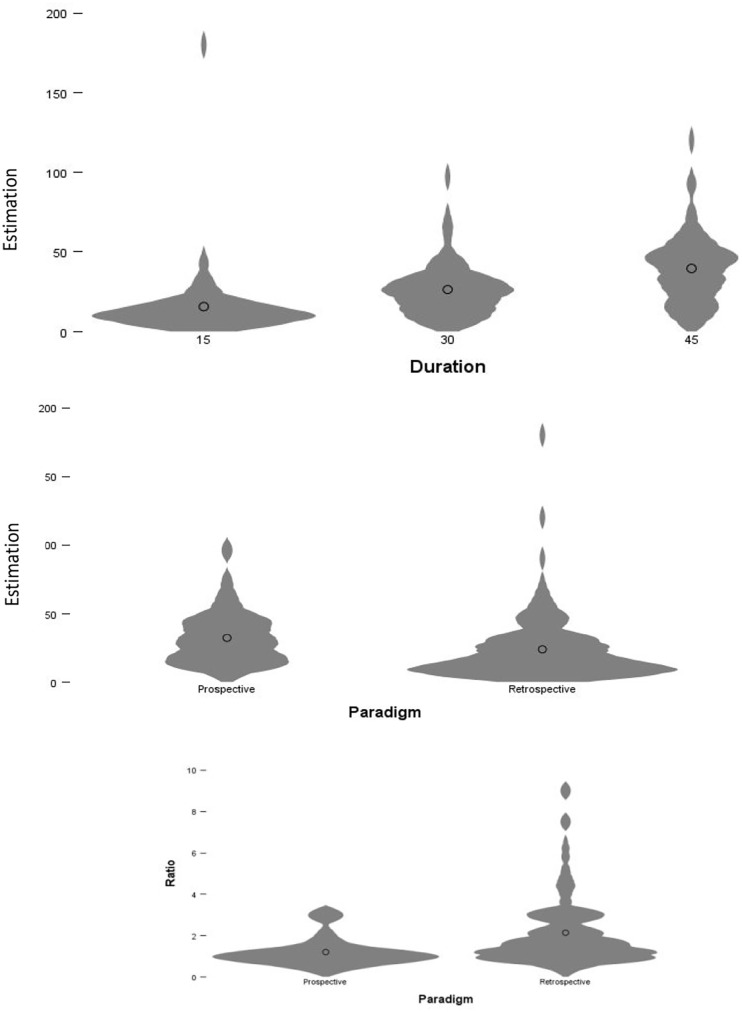
Results from Experiment 2. *Note.* In the top row (Panels 1 and 2, respectively), it can be seen that there was a main effect of duration which is to be expected and in accordance with Experiment 1. In Panel 2, we can see that there is a main effect of paradigm, in which subjects in the retrospective paradigm had an overall lower reproduction than subjects in the prospective paradigm. Finally, in the second row (or Panel 3), it can be seen that in terms of ratio, subjects were more accurate in the prospective – as opposed to retrospective – paradigm; the reverse of Experiment 1 as shown.

Similar to Experiment 1, the violin plots suggest that some variables might be skewed. Given that the sample for Experiment 2 (after cleaning) is approaching 200 (i.e. 195) the deviation of skew from normality will not make a substantive difference to the results (Tabachink & Fiddell, 2013). To test $H_{1}$ , the reproduction of subjects’ responses was analysed. A three-factor Between-Subjects ANOVA with paradigm (Retrospective/ Prospective) and schizotypy (Low/High) and duration (15 s, 30 s and 45 s) as between-subject factors. As indicated in Figure 4 (Panel 1), there was a significant main effect of duration, F(2,183)= $22.771,\;\;\;p<\;.001,\;\eta^{2}=\;.199,\;95\%\;\mathrm{CI}\;[0.1009,\;0.2918]$ and paradigm, as indicated in Figure 4 (Panel 2), *$F\left(1,\;183\right)=8.333,\;p=\;.004,\;\eta^{2}=\;.044,\;[0.0044,\;0.1134]$ *, however, as expected, there was no significant main effect of schizotypy, $F\left(1,\;298\right)=0.336,\;p=\;.563,\;\eta^{2}=\;.002,\;[0.0000,0.0333]$ , in accordance with Experiment 1. There were no significant interactions, (i.e. all $p_{s}>.05$ ). Given that the sensitivity analysis for Experiment 2 revealed that the SMSI was $\eta^{2}=.044$ , the main effects of duration $\left(\eta^{2}=.199\right)$ and paradigm $\left(\eta^{2}=.044\right)$ were above the threshold, implying the effects were detected despite the sample size.

The main effect of duration was further analysed by conducting Bonferroni-corrected post hoc *t*-tests, in which the alpha level was $\alpha =\frac{.05}{3}=.012$ . The mean difference ( M=−24.12) between the 15s(M=15.53;SD=22.54) and 45s(M=39.65) estimations was significant, *t* (125) = −6.077,p=<.001,d=−1.79,95%CI[1.45,−0.70] , as was the mean difference (M=−10.81) between the 15 s (M=15.53;SD=22.54) and 30s(M=26.34;SD=16.67) estimations, t(130)=−3.146,p=.001,d=−0.545,95%[−0.90,−0.20] . Finally, the mean difference (M=−13.31) between 30s(M=26.34;SD=16.67) and 45 s (*M* = 39.65; SD=22.19) estimations was also significant, t (129) = −3.901,p<.001,d=−0.682,[−1.03,−0.33] . This demonstrates, as expected that subjects perceived each of the durations as significantly different in accordance with Experiment 1 and Klapproth (2007).

To further analyse the main effect of paradigm, a Bonferroni-Corrected post-hoc t-test was conducted, in which the mean difference (M=8.39) between prospective (M=32.04;SD=19.35) and retrospective (M=23.65;SD=24.20) paradigms were significant, t(193)=2.580,p=.005,d=0.367,95% CI[0.09,0.663] , implying that subjects in the retrospective paradigm had lower overall reproductions.

The null hypothesis of $H_{1}$ thus can be rejected in that there were differences in terms of deviant reproductions in low and high schizotypy and the alternative hypothesis of $H_{1}$ can be accepted, in that there are no differences between low and high schizotypy, similar to Experiment 1. However, we note (also similar to Experiment 1) that subjects, overall, underestimated retrospective estimations as opposed to prospective estimations implying that different timing mechanisms are involved in both the prospective and retrospective paradigms.

To test $H_{2}$ , the ratio of reproductions, relative to actual durations, was taken in which answers closer to 1 indicate greater accuracy in reproducing the duration. As indicated by Figure 4 (Panel 3) there was a significant main effect of paradigm, $F\left(1,183\right)=23.242,p<.001,\;\eta^{2}=\;.113,\;$ 95%CI[0.0405,0.2017] but not of Schizotypy, F(1,183) $=0.160,p=.689.,\;\eta^{2}=.001,\;\left[0.0000,0.0279\right]$ or condition, $F\left(2,\;183\right)=0.386,\;p=.680,\;\eta^{2}=\;.001,\;$ [0.0000,0.0316] . There were no significant interactions, (i.e. all $p_{s}>.05$ ). Given the main effect of paradigm $\left(\eta^{2}=.113\right)$ , it is implied that this effect was robustly detected, in accordance with the sensitivity analysis.

We analysed the main-effect of paradigm by conducting a Bonferroni-Corrected Post Hoc *t*-test, in which the mean difference (M=−0.93) between the ratio of prospective (M=1.19;SD=0.67) and retrospective (*M* = 2.12; SD=1.71) was significant t(193)=−4.650,p<.001, d=−0.677,95%CI[−0.97,−0.38] implying that subjects were more accurate in the prospective paradigm, as illustrated in Figure 4 (Panel 3). Overall, we can accept the alternative hypothesis for $H_{2}$ in that high schizotypy subjects’ accuracy is contextually bounded, given that the stimulus used in Experiment 2 was contextually neutral. However, interestingly, the main effect of paradigm is opposite to the finding in Experiment 1 however, since the stimulus in Experiment 1 was contextualized, as opposed to the stimulus in Experiment 2, this is to be expected.

To test the final hypothesis $\left(H_{3}\right)$ , the intersubject variability of subjects’ responses was tested. There were no significant main effects (i.e. all $p_{s}>.05$ ) or interactions (i.e. all $p_{s}>.05$ ), meaning that we can accept our final hypothesis $H_{3}$ given that, as expected, there were no differences between high and low schizotypy on intersubject variability.

Therefore, we have accepted our main hypothesis $\left(H_{2}\right)$ that high schizotypy subjects’ duration reproductions are driven by the pacemaker/accumulator model. Consequently, the results of both Experiments 1 and 2 lend support to the idea that schizophrenic subjects (and possibly, schizotypy) are more accurate in perceiving a concrete stimulus as opposed to a neutral stimulus (Ducato et al., 2008). Therefore, based on the results of Experiments 1 and 2, we shall argue that timing deficits in high schizotypy are driven by the context of the stimulus used, which is similar to schizophrenia demonstrating a similar aetiology of both schizophrenia and schizotypy, respectively, at least in the context of the perception of time.

## General discussion

Overall, we report the results of a two-study investigation in exploring whether timing deficits in schizotypy (and, by extension, schizophrenia) are driven by the attentional mechanisms of the pacemaker/accumulator model. To test this assumption, we conducted two experiments: the first experiment utilized a contextually heavy stimulus (i.e. a video of kittens partaking in play); while the second experiment utilized a contextually neutral stimulus (i.e. the River Nene in Northampton, UK). Overall, our findings suggest that schizotypy timing is attentionally bounded within the context of the pacemaker/accumulator component of SET, as evidenced by Experiments 1 and 2, respectively. Furthermore, we suggest that these findings can be mapped onto schizophrenia to further disentangle the mechanistic properties of the perception of time in schizophrenic subjects.

In terms of the findings of Experiment 1, several key findings emerged. For the analysis of estimations, it was shown that high schizotypy subjects did not underestimate durations, despite there being a trend implying as such, meaning that $H_{1}$ was rejected. Though, it was found that subjects underestimated the retrospective durations, overall. was confirmed, in which high schizotypy subjects showed variability in ratio values, of which it was shown they were more accurate, irrespective of duration and paradigm. However, it was also the case that, overall, subjects were more accurate in the retrospective paradigm than the prospective paradigm. Finally, $H_{3}$ was rejected, given that there was no interaction between schizotypy, duration and paradigm. However, there was a main effect of paradigm, demonstrating less variability in the retrospective paradigm, as opposed to the prospective paradigm. Interestingly, the interaction between paradigm and duration showed that subjects had less variability in the retrospective 15 s and 45 s, as opposed to the 30 s duration. Experiment 1 formed the basis of Experiment 2, and the hypotheses were formulated accordingly.

In terms of Experiment 2, we further explored whether high schizotypy were more accurate in Experiment 1 due to a highly contextualized stimulus (i.e. a kitten video). Therefore, we replicated Experiment 1 in all, but the stimulus used (i.e. in Experiment 2, we used a neutral stimulus of the River Nene in Northampton, UK). Several findings emerged: we hypothesized that given the neutral features of the stimulus in Experiment 2 that high schizotypy would not show any significant differences compared to low schizotypy with respect to reproductions. $H_{1}$ was accepted in that we found no differences between low and high schizotypy. Similar to Experiment 1, we found that there was a main effect of paradigm, in which subjects appeared to underestimate retrospective conditions relative to prospective conditions. $H_{2}$ was also confirmed in that high schizotypy was no more accurate in recognising the duration than low schizotypy however, we also found evidence for an opposed finding in that the main effect of paradigm, in which this time, subjects were less accurate in recalling retrospective paradigms. Finally, $H_{3}$ was confirmed in that there were no differences between low and high schizotypy, with respect to intersubject variability. Each of these findings shall be discussed in accordance with SET, as well as briefly considering these findings with respect to neural oscillations (Treisman et al., 1994) driving the pacemaker/accumulator component.

In both Experiments 1 and 2, there was no evidence of high schizotypy demonstrating timing deficits relative to low schizotypy. It was hypothesized, at least for Experiment 1 that high schizotypy would demonstrate deficits in reproducing durations, given that in some tasks (i.e. Carroll et al., 2008), schizophrenics underestimate durations, and in others high schizotypy overestimate durations (i.e. Reed & Randell, 2014). Given that prospective timing is said to be driven by both attentional and working memory processes, which accords to the pacemaker–accumulator and working memory components of SET (Klapproth, 2007; Gibbon, 1977), the hypothesis for Experiment 1 argued that high schizotypy subjects would underestimate durations, given working memory deficits (Reed & Randell, 2014; Haigh et al., 2022; Huang et al., 2021) however, there is evidence that schizophrenic subjects pay more attention to concrete stimuli (Ducato et al., 2008) in low-attentional tasks (i.e. such as a kitten video). This implies that the lack of a deficit in the pacemaker–accumulator model of schizotypy is due to high schizotypy paying more attention to concrete stimuli if the aetiology of schizophrenia and schizotypy is similar (Lenzenweger, 2006), compensating for any perceived deficits in pacemaker/accumulator. In the context of neural oscillators (Treisman et al., 1994) mechanism for pacemaker/accumulator models, it has been argued that general timing is driven by numerous oscillators in the alpha range (8−13MHz) , in which faster alpha rates are associated with overestimation of duration, and low alpha rates are associated with an underestimation of durations. Given that it has been argued that subjects with high schizotypal traits often show lower alpha frequencies than subjects without schizotypal traits (Ippolito et al., 2022), this would imply that high schizotypy should have underestimated durations however, this is not the case in our study, implying the neural oscillations driving the pacemaker/accumulator model are not aberrant in schizotypy. A further finding that emerged from testing $H_{1}$ in both Experiments 1 and 2 is the difference between prospective and retrospective paradigms, in direct contrast to Klapproth (2007), who showed that there were no differences between prospective and retrospective paradigm. This study presents evidence that there are different timing mechanisms for prospective and retrospective paradigms, which accords with research by Zakay and Block (1998), who argued that when subjects make retrospective judgements, they do so on the basis of nontemporal information within the duration and the complexity of the stimuli (Block, 1989; Ornstein, 1969), which implies that the stimulus subjects were exposed to influenced their judgement of the duration in the retrospective paradigm, given that subjects underestimate past events (Roy et al., 2005) and generally, underestimate retrospective durations (Ei Haj et al., 2013). Therefore, we explain the effect found in the retrospective paradigm due to subjects utilizing a different timing mechanism, to prospective durations, and underestimating durations, given humans’ propensity to underestimate past events. There is further evidence for this in the testing of $H_{2}$ .

The finding that $H_{2}$ was confirmed in Experiment 1, and that high schizotypy subjects were more accurate in identifying durations feeds on directly from the explanation in the previous paragraph. Given that the stimulus used was contextually complex (i.e. a kitten video), and that schizophrenic subjects pay more attention to concrete stimuli, it could be argued that high schizotypy subjects had a better representation of the duration in visual working and reference memories and could therefore readily identify the stimulus duration more accurately than low schizotypy. Therefore, at least in the prospective paradigm, given that high schizotypy subjects were able to inhibit prepotent responses to the stimuli, and pay full attention to the duration of the stimuli (c.f., Yücel et al., 2002); whilst in the retrospective paradigm – and working within the framework of SET, it can be argued that given that high schizotypy subjects show superior attention in attentional resources, when the pacemaker–accumulator model accumulated time pulses, their representation in working memory was more accurate than in low schizotypy subjects therefore, in terms of comparing this to a duration in reference memory, high schizotypy subjects had an advantage in doing so given that the stimulus was more concrete. This is further collaborated by Experiment 2, in which a neutral-context stimulus was used, and led to the effect of the superior accuracy of high schizotypy disappearing, implying that given that the neutral-context stimuli were not concrete, the attentional-driven pacemaker–accumulator model did not have a better representation of the duration, thereby extinguishing the effect of greater attentional resources. Within the context of the neural pacemaker oscillator, it has been argued that more accurate temporal perception of visual stimuli is associated with a quicker alpha frequency peak (Marcicano et al., 2022) however, there is further evidence that schizotypy subjects have less temporal acuity than baselines, especially in the context of the Temporal Binding Window (Fenner et al., 2020), in which the subject integrates multiple stimuli into a single event. However, given that in our study, High Schizotypy did not show aberrations in the pacemaker, compared to Low Schizotypy, it can be argued that, at least in this study, schizotypy is not associated with less temporal acuity – indeed, an opposite effect was reported on the basis of context, in which high context scenes (i.e. Experiment 1) led to high Sshizotypy reporting greater levels of temporal acuity. However, a further finding that emerged whilst testing $H_{2}$ in both Experiments 1 and 2 was that subjects were more accurate in identifying retrospective durations, as opposed to prospective durations in Experiment 1. This accords with the older literature (i.e. Hintzman & Block, 1971) who showed that subjects can make accurate temporal dating judgements – however, Zakay and Block (1996) also argued that retrospective durations are driven more by the context of the stimuli, and given that in Experiment 1, the stimulus used was a kitten video, this finding accords with the available evidence. This finding is further collaborated in Experiment 2. In which the finding was opposite from that of Experiment 1, in which subjects were less accurate in identifying retrospective durations than prospective durations, which is to be expected, given the neutral nature of the stimulus used in Experiment 2. Therefore, we argue that our findings across both experiments imply there are different timing mechanisms for each paradigm (Zakay & Block, 1996) and that, in accordance with Zakay and Block (1997) retrospective accuracy is dependent on the context of the stimuli. Therefore, we explain the finding that subjects were more accurate in identifying retrospective durations, in Experiment 1, given the fact that the context of the stimulus is of greater importance to retrospective durations. This is further collaborated by the finding in Experiment 2 that this effect is reversed when using neutral stimuli.

The final hypothesis to be tested $\left(H_{3}\right)$ was whether high schizotypy would show greater intersubject variability than low schizotypy subjects. Once again, the hypothesis was rejected in that high schizotypy did not show any differential differences in intersubject variability, compared to controls. This can be explained, again, by the fact that high schizotypy has a greater degree of attention dedicated to concrete stimuli than low schizotypy, implying that duration judgement will also be less variable. This finding can be explained in terms of the ‘switch’ component in SET. Emitted pulses by the pacemaker must pass through an attentional switch (Zakay & Block, 1996) prior to accumulation. When this switch is ‘closed’ the pulses can accumulate without variability, meaning that there is an accurate representation of durations in the accumulator, ready to be transferred to working memory for comparison. Given our findings that high schizotypy shows an overall advantage in attention, it can be argued that their switch is less variable and therefore, duration judgements will be less variable overall. However, an interesting finding that arose whilst testing $H_{3}$ is that there was less variability in retrospective 15 s and 45 s than Prospective equivalents, but not in 30 s, implying that subjects, overall, were less variable in the shortest and longest of the durations, but not the mean. This can be explained, via analogy with the temporal bisection task (Wearden et al., 1996). In such a task, subjects typically have <5% long responses for ‘Short’ and >90% long responses for Long’ durations however, for the mean value of these (i.e. say 200 ms is the short and 800 ms is the long, the mean would be 500 ms), subjects show greater variability in conflating the mean value, which appears to have occurred in these data hence why the intersubject variability between the 30 s retrospective and prospective paradigm was not significant. In Experiment 2, there were no main effects or interactions, demonstrating that the context of the stimuli plays a role in intersubject variability.

Thus, the main crux of this study is the following: (a) that high schizotypy accuracy in timing is driven by attentional deficits though, as opposed to a deficit in the traditional sense (i.e. on a negative axis); the deficit gives high schizotypy improved accuracy. We showed in Experiment 1 that high schizotypy was more accurate in identifying durations, given the contextual nature of the kitten video. However, in Experiment 2, this effect disappeared given that the stimuli were neutral. The other major finding of this study is that there appear to be different timing mechanisms driving both Prospective and Retrospective timing, as evidenced by Experiments 1 and 2, respectively.

Despite these novel findings (i.e. that the perception of time appears to be driven by the context of the stimulus in high schizotypy), further research should investigate whether this finding is found in baseline and schizophrenic conditions, using a similar paradigm. Given that it is argued that schizotypy and schizophrenia share a similar aetiology, we would argue that such a finding should be found in schizophrenia. Another potential avenue to explore is whether context can be used to correct deviant perception of time in schizophrenia. Evidence suggests that the perception of time can be modified by a click train (Wearden et al., 1996); which coupled with the results here, implies that the perception of time in schizophrenia could be moderated by having a context-rich stimulus.

In terms of the limitation of this study, one potential issue is a lack of an auditory equivalent to the visual task used. This would allow researchers to fully test the enhanced visual memory component in schizotypy and potentially schizophrenia. A further limitation was that due to the online nature of the experiment (i.e. using the Gorilla.sc software, as data were collected during the COVID-19 era), it was not possible to include an attentional check. Therefore, it is possible that subjects could have timed the durations (especially in the prospective paradigm) or they were not paying their full attention to the study. Therefore, any future studies should contemplate running both a visual and auditory analogue of this task and also, ensure that an attentional check is used in future studies, if an online experiment is utilized in future experiments.

In summary, across two experiments, we present evidence that high schizotypy (and possibly, schizophrenics) are more accurate in identifying judgement duration due to the contextual bases of the stimulus (Ducato et al., 2008). This investigation implies that the pacemaker/accumulator model in schizophrenia seems to be affected by the stimulus. Further investigation is required – particularly in clinical populations – to ascertain this result.
